# The global status of research in prostate cancer bone metastasis: A bibliometric and visualized analysis

**DOI:** 10.3389/fmed.2022.931422

**Published:** 2022-08-03

**Authors:** Zongwei Lv, Xia Wang, Chunming Zhu, Kefeng Wang

**Affiliations:** ^1^Department of Urology, Shengjing Hospital of China Medical University, Shenyang, China; ^2^Department of Family Medicine, Shengjing Hospital of China Medical University, Shenyang, China

**Keywords:** bibliometrics, prostate cancer, bone metastasis, prognosis, diagnosis

## Abstract

**Background:**

Prostate cancer (PCa) is a serious threat to the health of elderly aged groups. It is very important to understand the occurrence and development of PCa for early diagnosis, treatment and metastasis control. This study aims to elucidate the international frontier research direction and literature distribution through bibliometric and visual analyses of PCa bone metastasis.

**Methods:**

Data were obtained from the Web of Science core collection database, which collected 2,246 papers related to PCa bone metastasis from 1 January 2012 to 31 December 2021. The collected data were analyzed using the VOSviewer software for citation, co-authorship, co-citation, bibliometric coupling, and co-occurrence.

**Results:**

Over the past decade, published papers have increased annually. The United States of America has published 890 papers with 29,161 citations, far more than any other country, and it has the most extensive collaboration with other countries. For example, 33 articles by Saad Fred were cited 2,721 times, and 91 articles from the University of Texas MD Anderson CANC CTR were cited 3,037 times, the most cited author and organization. Peng Xinsheng and Duke UNIV comprise the most active collaborative author and organization, respectively. The most co-cited journal was CANCER RES, with 3,195 citations. Studies of PCa bone metastasis can be divided into four categories: “basic research,” “auxiliary diagnosis and treatment,” “clinical trial,” and “prognosis.”

**Conclusion:**

Our results provide a comprehensive overview of the research priorities and future directions of PCa bone metastasis, which can further accurately guide researchers in diagnosis, treatment, and personalized prevention.

## Introduction

In 2020, a total of 1.41 million new cases of prostate cancer (PCa) were reported globally (1), and PCa remains one of the most common malignancies in men (2). According to a cancer statistics, in 2022, the United States of America (United States) will have an estimated 268,490 cases of PCa, accounting for 27% of male malignancies, which is higher than that of lung and bronchial cancers, which rank second, and 34,500 PCa-related deaths, which accounts for second highest among male malignancies. Therefore, PCa seriously affects the health and quality of life of older men (3). With medical advancement, the 5-year survival rate of patients with PCa has reached more than 70% in most countries and as high as 90% in some countries, with many countries showing an increase in the annual survival rate (4). However, distant metastasis, including bone, lymph node, lung, and liver metastases, is still the main cause of death in patients with PCa (5). Bone metastasis is the most common site for advanced PCa (6). Once bone metastasis occurs, it often causes various bone-related events, and the clinical manifestations are bone pain, pathological fracture, nerve compression syndrome, and hypercalcemia, which often lead to poor prognosis for patients with PCa (7).

Bibliometrics is a method that combines mathematical and statistical methods. It provides statistics of research results, performs quantitative analysis of literature using mathematical methods, analyses key areas of research, understands the quality of research, and predicts future research directions (8, 9). Bibliometric analysis plays an important role in diagnosing and treating diseases and formulating clinical guidelines. They are widely used and play an important role in the biomedical field. For example, Chen et al. (10) found that bibliometric analysis helps obtain the distribution characteristics of the literature through statistical analysis. It helps researchers find research partners, optimize research topics, and monitor new scientific and technological activities. Shi et al. (11) summarized the main treatment therapies through bibliometric analysis of breast cancer liver metastasis, which was of great significance.

Recently, Shen et al. (12) collected more than 20 years of literature and published a global bibliometric analysis of artificial intelligence in PCa. In addition, Shi et al. (11) conducted a bibliometric evaluation of liver metastasis in breast cancer. However, no bibliometric articles on PCa bone metastasis have been published. Therefore, to the best of our knowledge, our study is novel and provides the latest information for understanding the status of PCa bone metastasis research worldwide.

Recently, many scientific achievements have been made in the study of PCa bone metastasis. Bibliometric analysis can be used to analyze the literature published in this field over the last decade. Using VOSviewer software, the collaboration between authors, organizations, and countries/regions were deeply explored, while the keywords, journals, and references were comprehensively analyzed (11). Through bibliometric and visual analyses, the current research focus on PCa bone metastasis is understood, guiding the future diagnosis and treatment of PCa bone metastasis. Our study will help elderly male patients to have a sufficient understanding of the disease, providing some help for aging and prolonging human life.

## Materials and methods

### Data source and search strategy

**FIGURE 1:**
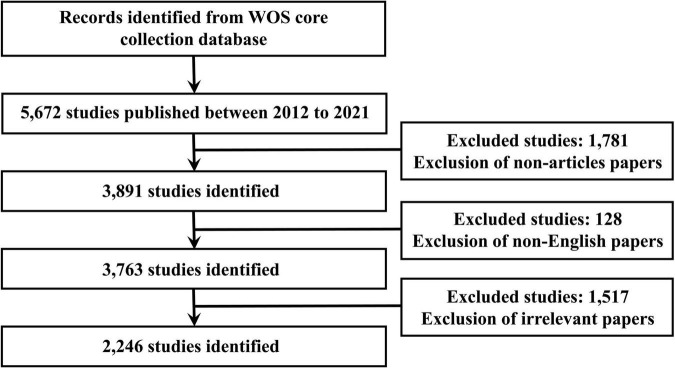
Flow chart of literature screening related to PCa bone metastasis research.

Global literature on PCa bone metastasis was searched in the Web of Science (WOS) core collection database from 1 January 2012 to 31 December 2021. The search strategies were as follows: TS = [(“prostate cancer” OR “prostate carcinoma”) AND (“bone metastas*” OR “skeletal metastas*”)] OR TS = “prostate cancer bone metastas*” OR TS = “bone metastas* from prostate cancer.” The types of documents were limited to articles, and the language was limited to English. Detailed data retrieval and inclusion procedures are shown in Figure 1.

### Data collection and analysis

The file information was downloaded from the WOS core collection database. Full records and cited references were obtained. The documents were downloaded in a plain text format. VOSviewer 1.6.18 software was used to comprehensively analyze the collaborative relationships between authors, organizations, countries/regions, literature coupling indicators, keywords through citation, co-authorship, co-citation, co-occurrence, and bibliometric coupling. The maps were presented through a network, overlay, and density visualization (13). Different colors expressed different clusters in the network visualization. In all the visualization map, color generated has certain rules. It is based on the number of different clusters to produce the particular color. The color of the largest cluster (cluster 1) is red; the second largest cluster (cluster 2) is green in color; the third largest cluster (cluster 3) is blue in color; the fourth largest cluster (cluster 4) is yellow in color; the color of following clusters are purple, cyan, orange and so on. In all the overlay visualization map, it is based on the average year of publications or keywords to produce the particular color. The color changes gradually from purple to yellow as the average year grow.

Microsoft Excel 2019 was used to analyze and plot the top 10 most active authors, organizations, and countries/regions related to PCa bone metastasis, including the number of published papers and citations. In addition, the top 10 co-cited references related to PCa bone metastasis, including authors, countries, publication years, and corresponding journals, are described in an Excel table. The annual number of publications and the top 10 published and cited journals were also analyzed and drawn.

## Results and discussion

### Bibliometric analysis of publication output

**FIGURE 2:**
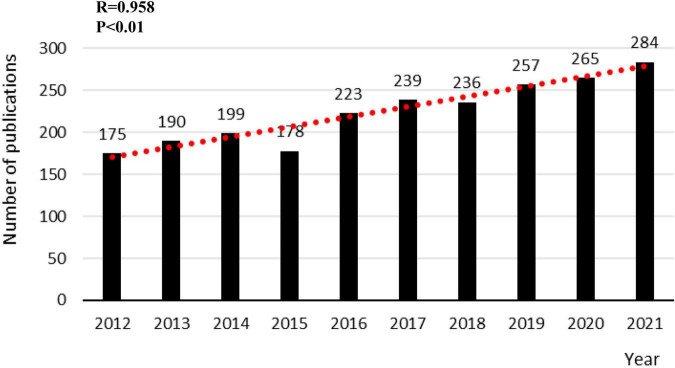
The annual number of publications on PCa bone metastasis from 2012 to 2021.

Through screening, 2,246 publications related to PCa bone metastasis were acquired from the WOS core collection database. The number of papers published yearly is shown in Figure 2. The figure shows that the highest number of published papers was 284 in 2021 and the lowest was 175 in 2012. Over the past decade, there has been a general upward trend in the number of publications. This indicates that there are more focuses and hotspots worth exploring in PCa bone metastasis and that the research prospects will be brighter.

### Bibliometric analysis of the publications and citations

**FIGURE 3:**
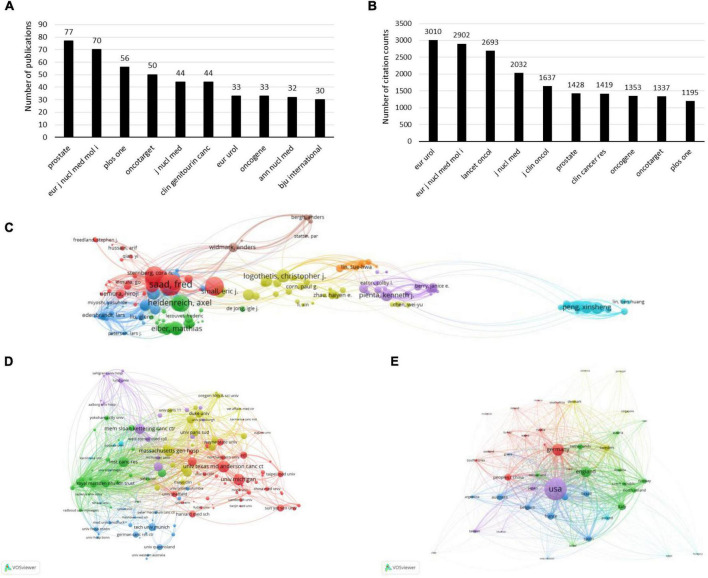
The bibliometric analysis of the publications and citations on PCa bone metastasis. **(A)** The top 10 published journals in the field of PCa bone metastasis. **(B)** The top 10 cited journals in the field of PCa bone metastasis. **(C)** The bibliometric analysis of authors in the field of PCa bone metastasis. **(D)** The bibliometric analysis of institutions in the field of PCa bone metastasis. **(E)** The bibliometric analysis of countries/regions in the field of PCa bone metastasis.

A total of 579 journals published papers on PCa bone metastasis, including 111 journals with more than five papers. The top 10 published and cited journals are plotted in Figures 3A,B. The figure shows that the journal that published the most papers was Prostate, with 77 papers and an impact factor (IF)/journal citation reports (JCR) partition of 4.104/Q3 in 2021. The journal with the most cited papers was EUR Urol, with 3,010 citations and an IF/JCR partition of 20.096/Q1 in 2021. EUR J Nucl Med Mol I published 70 papers and was cited 2,902 times, ranking second in publications and citations. The IF/JCR partition was 9.236/Q1 in 2021.

VOSviewer software was used to conduct a network visualization analysis of the citations for authors, institutions, and countries/regions, as shown in Figures 3C–E. The top 10 most active authors, organizations, and countries/regions of PCa bone metastasis are listed in Tables 1–3. As shown in Figure 3C, the author who was cited the most (2,721 times) was Saad Fred, who published 33 papers on PCa bone metastasis. As shown in Figure 3D, the University of Texas MD Anderson CANC CTR published 91 papers with 3,037 citations, making it the most cited organization. In Figure 3E, the United States produced far more papers and citations than any other country. In the past decade, 890 papers have been published in the United States, concerning PCa bone metastasis, with 29,161 citations. The total link strength was 9,312.

**TABLE 1:** The top 10 most active authors related to PCa bone metastasis.

Rank	Name	Number of publications	Count of citations
1	Saad, fred	33	2,721
2	Pienta, kenneth j.	29	1,115
3	Logothetis, christopher j.	27	1,208
4	Peng, xinsheng	24	1,094
5	Fizazi, karim	23	2,145
6	Tombal, bertrand	23	1,374
7	Ren, dong	21	1,038
8	Huang, shuai	21	830
9	Uemura, hiroji	21	740
10	Keller, evan t.	21	672

**TABLE 2:** The top 10 most active organizations related to PCa bone metastasis.

Rank	Name	Number of publications	Count of citations
1	Univ texas md anderson canc ctr	91	3,037
2	Univ michigan	79	2,963
3	Mem sloan kettering canc ctr	59	2,518
4	Univ washington	59	1,945
5	Johns hopkins univ	48	1,973
6	Duke univ	47	1,669
7	Inst canc res	44	2,425
8	Sun yat sen univ	42	1,299
9	Cedars sinai med ctr	41	1,080
10	Univ calif los angeles	39	1,260

**TABLE 3:** The top 10 most active countries/regions related to PCa bone metastasis.

Rank	Name	Number of publications	Count of citations
1	United States	890	29,161
2	Peoples r china	344	5,508
3	Germany	241	12,100
4	England	190	10,182
5	Japan	174	3,233
6	Italy	162	5,916
7	Canada	145	5,489
8	Sweden	103	5,106
9	Australia	101	4,772
10	France	96	6,011

### Bibliometric analysis of the co-authorship

#### Co-authorship analysis of authors

**FIGURE 4:**
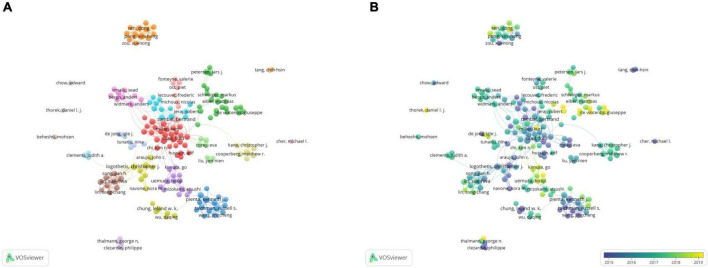
The bibliometric analysis of the co-authorship of authors in the field of PCa bone metastasis. **(A)** The network visualization map of authors collaboration in the field of PCa bone metastasis. **(B)** The overlay visualization map of authors collaboration in the field of PCa bone metastasis.

A total of 179 authors with more than seven publications were selected for the co-authorship analysis. In the network visualization map (Figure 4A), Peng Xinsheng (China) was found to collaborate with other authors frequently, mainly Ren Dong (China) and Guo Wei (China), forming a small research group. In 2017, they published a paper titled “Oncogenic miR-210-3p promotes PCa cell EMT and bone metastasis *via* the NF-κB signaling pathway.” They identified a novel activation mechanism of the NF-κB signaling pathway, closely associated with PCa bone metastasis. These results suggested that epigenetic events play an important role in PCa bone metastasis (14). In addition, many authors have collaborators, and different colors represent different groups of collaborators. For example, other groups of researchers, including Lin Suehwa (United States), Lee yu Liyuan (United States), Lee Yuchen (Taiwan, China), Yu Guoyu (United States), and Lin Songchang (United States), have also produced many academic results. They identified a mechanism that can induce the dormancy of diffuse tumor cells through the TGF-β RIII-P38MAPK-PS249/PT252-Rb signaling pathway, which provided a theoretical basis for the development of guidelines to prevent PCa recurrence (15). Some authors, such as Beheshti Mohsen and Cher Michael I, focused on individual studies without forming teams. It was observed that the collaborative network of individual authors was decentralized in PCa bone metastases.

In the overlay visualization map (Figure 4B), it was noticed that Monari Fabio (Italy), Ilhan Harun (Germany), and Tilki Derya (Germany) have been the authors of interest in PCa bone metastasis in recent years. They had their own partners and were conducting their research.

#### Co-authorship analysis of organizations

**FIGURE 5:**
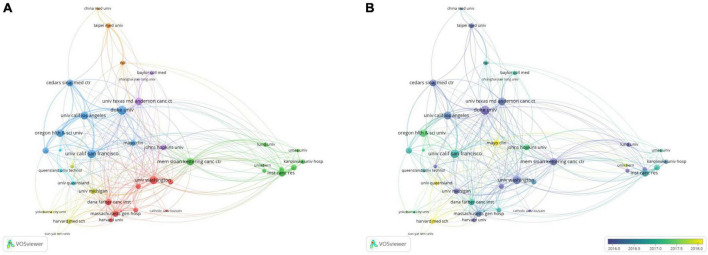
The bibliometric analysis of the co-authorship of organizations in the field of PCa bone metastasis. **(A)** The network visualization map of collaborations among organizations in the field of PCa bone metastasis. **(B)** The overlay visualization map of collaborations among organizations in the field of PCa bone metastasis.

A total of 41 organizations that published more than 20 papers were selected for the co-authorship analysis (Figure 5A). The organizational cooperation network had an overall and regional concentration degree. Duke UNIV (United States) had collaborative relationships with 26 organizations, making it the most active organization. It has published 47 related papers, with 1,669 citations and a total link strength of 108. The main partners of this organization were the Oregon HLTH and SCI UNIV (United States), UNIV California Los Angeles (United States), and UNIV California San Francisco (United States). These well-known organizations have formed an influential research team in PCa bone metastasis. One study showed that dasatinib could treat castration-resistant prostate cancer (CRPC) with bone metastasis. The results also showed a correlation between efficacy and progression-free survival (PFS) (16). In addition, MEM Sloan Kettering CANC CTR (United States), in collaboration with Lund UNIV (Sweden), developed an automatic quantification of the bone scan index (BSI) and identified its clinical significance. The automatic BSI score eliminates the operator’s subjective dependence, which is reproducible and can provide more important clinical information than the manual BSI score (17). These results demonstrate the importance of collaboration between institutions. The UNIV Texas Ed Anderson CANC STR (United States), which published the most papers, was associated with 27 organizations. However, the total link strength was only 74, ranking sixth. One of the important reasons for the low ranking of the total link strength is the lack of collaboration with other organizations.

It was discovered from the overlay visualization map (Figure 5B) that the three organizations were most interested in PCa bone metastasis after 2018. Harvard Med SCH (United States) was the most popular institution, followed by Mayo Clinic (United States) and UNIV Queensland (Australia). Harvard Med SCH published 23 papers on PCa bone metastasis and was interested in the basic research and treatment of PCa bone metastasis. One of their studies showed that upregulation of miR-582-3p and miR-582-5p inhibited the invasion and migration abilities of PCa cells *in vitro* and repressed bone metastasis *in vivo* by repressing TGF-beta signaling. This suggests its potential therapeutic value for treating PCa bone metastasis (18). In addition, the results of clinical trial in Harvard Med SCH showed that Radium-223 was well tolerated in combination with docetaxel in a Phase 2A trial. The combination enhanced antitumor activity compared to docetaxel alone (19). These findings of Harvard Med SCH are of great significance for the treatment of PCa bone metastasis in upregulating related miRNA and drug therapy.

#### Co-authorship analysis of countries/regions

**FIGURE 6:**
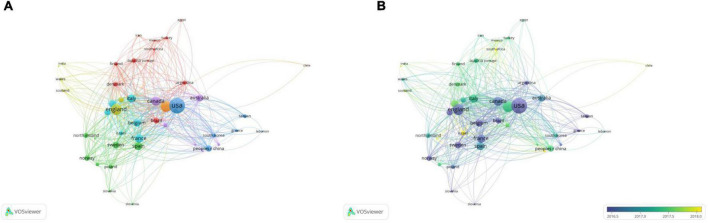
The bibliometric analysis of the co-authorship of countries/regions in the field of PCa bone metastasis. **(A)** The network visualization map of countries/regions collaborations in the field of PCa bone metastasis. **(B)** The overlay visualization map of countries/regions collaborations in the field of PCa bone metastasis.

A total of 46 countries/regions with a publication frequency of more than five times were selected for network visualization analysis (Figure 6A). The United States had the highest production and extensive collaboration with other countries. In the past 10 years, 890 papers and 853 link strengths have been published on PCa bone metastasis. The United States has links to 45 countries, with China and Germany having the largest partners, both with 76 link strengths, followed by England and Canada. The paper “Alpha Emitter Radium-223 and Survival in Metastatic Prostate Cancer,” published in the New England Journal of Medicine in 2013 by England, the United States, Sweden, Norway, and other countries, was cited 1,899 times and continues to be considered the most popular article. The efficacy and safety of radium-223 in patients with CRPC and bone metastasis were evaluated in this study. Clinical trials have demonstrated that radium-223 improves the overall survival (OS) of patients (20). France published only 96 papers, however, its total link strength was higher than that of Italy, which published 162 papers. This result may be because France has published several highly cited articles on PCa bone metastasis in collaboration with other countries, leading to a high total link strength. Although Italy published more articles, the degree of collaboration with other countries was low; thus, the total link strength was lower than that of France. French and German scientists have collaborated to discover the location and function of the gap junction protein Cx43 in mice, which may play a decisive role in PCa bone metastasis (21). Therefore, academic cooperation between countries is crucial, and mutual exchange can promote innovation and development.

From the overlay visualization map (Figure 6B), the United States, Italy, Canada, and other countries were the first to study PCa bone metastasis. Since 2018, only four countries, Israel, Hungary, South Africa, and New Zealand, have shown an interest in PCa bone metastasis. There is a downward trend in cooperation between countries.

### Bibliometric analysis of the co-citation

**FIGURE 7:**
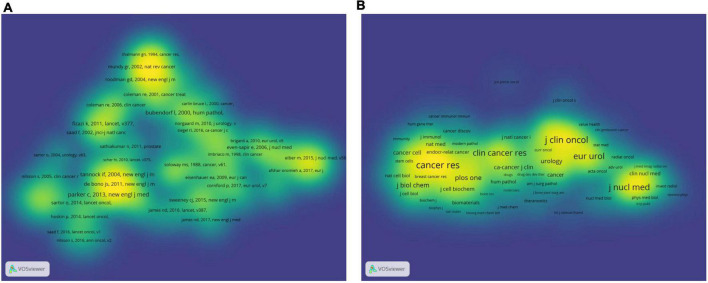
The bibliometric analysis of the co-citation in the field of PCa bone metastasis. **(A)** The density visualization map of co-cited references in the field of PCa bone metastasis. **(B)** The density visualization map of co-cited journals in the field of PCa bone metastasis.

The co-cited references have an important dynamic relationship. It reveals the development status and changes in research in related fields and can be used for frontier analysis, field analysis, and scientific research evaluation. In addition, co-citations can provide advanced support and a theoretical basis for scientific decision-making (10, 22). In this study, VOSviewer was used for co-citation analysis to screen out references that were co-cited more than 30 times. A total of 134 papers were selected for the density visualization map (Figure 7A). As shown in Table 4, a table of the top 10 co-cited references was created, including article titles, corresponding authors, countries, journals, and total citations. The most co-cited article was published by Parker C in New Engl J Med in 2013, with 276 co-citations, followed by Bubendorf L and Tannock IF. Five countries were among the top ten co-cited references, including the United States, the United Kingdom, Canada, France, and Switzerland.

**TABLE 4:** The top 10 co-cited references related to PCa bone metastasis.

Rank	Article title	Author	Country	Year	Journal
1	Alpha emitter radium-223 and survival in metastatic prostate cancer	Parker C	United Kingdom	2013	N Engl J Med
2	Metastatic patterns of prostate cancer: an autopsy study of 1,589 patients	Bubendorf L	Switzerland	2000	Hum Pathol
3	Docetaxel plus prednisone or mitoxantrone plus prednisone for advanced prostate cancer	Tannock IF	Canada	2004	N Engl J Med
4	Design and end points of clinical trials for patients with progressive prostate cancer and castrate levels of testosterone: recommendations of the Prostate Cancer Clinical Trials Working Group	Scher HI	United States	2008	J Clin Oncol
5	Denosumab versus zoledronic acid for treatment of bone metastases in men with castration-resistant prostate cancer: a randomized, double-blind study	Fizazi K	France	2011	Lancet
6	Increased survival with enzalutamide in prostate cancer after chemotherapy	Scher HI	United States	2012	N Engl J Med
7	Abiraterone and increased survival in metastatic prostate cancer	de Bono JS	United Kingdom	2011	N Engl J Med
8	Prednisone plus cabazitaxel or mitoxantrone for metastatic castration-resistant prostate cancer progressing after docetaxel treatment: a randomized open-label trial	de Bono JS	United Kingdom	2010	Lancet
9	Metastasis to bone: causes, consequences and therapeutic opportunities	Mundy GR	United States	2002	Nat Rev Cancer
10	Chemohormonal Therapy in Metastatic Hormone-Sensitive Prostate Cancer	Sweeney CJ	United States	2015	N Engl J Med

As observed from the density visualization map of journals (Figure 7B), there were six journals whose source density was much higher than that of other journals, and their co-citation times were more than 2,000 times. Among these, CANCER RES had the most co-cited sources, with a total co-cited time of 3,195 and an IF/JCR partition of 12.701/Q1 in 2021. The other five journals were J Clin ONCOL with a total co-cited time of 2,788 and an IF/JCR partition of 44.544/Q1 in 2021, EUR Urol with a total co-cited time of 2,499 and an IF/JCR partition of 20.096/Q1 in 2021, J Nucl Med with a total co-cited time of 2,450 and an IF/JCR partition of 10.057/Q1 in 2021, New Engl J Med with a total co-cited time of 2,189 and an IF/JCR partition of 91.245/Q1 in 2021, and Clin Cancer Res with a total co-cited time of 2,111 and an IF/JCR partition of 12.531/Q1 in 2021.

### Bibliometric analysis of the bibliographic coupling

**FIGURE 8:**
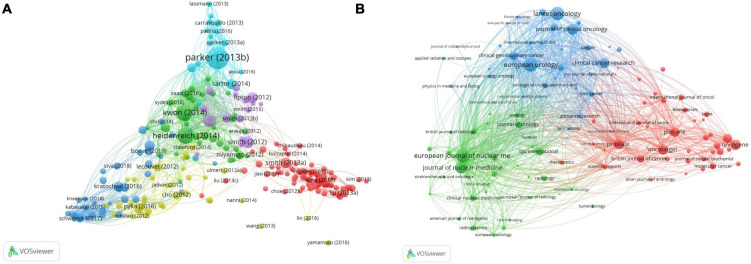
The bibliometric analysis of the bibliographic coupling in the field of PCa bone metastasis. **(A)** The network visualization map of bibliographic coupling of articles in the field of PCa bone metastasis. **(B)** The network visualization map of bibliographic coupling of journals in the field of PCa bone metastasis.

Bibliographic coupling is a static relationship in the literature (23). The minimum number of citations in the bibliographic coupling screening was 50. A total of 234 projects were included in the six clusters (Figure 8A). The main research areas were basic research (red), followed by diagnosis and treatment (dark blue), and clinical trials (green). Of a total of 234 projects, 87 projects are represented in red, which is the largest and far higher than the other clusters.

According to the network visual analysis of the sources (Figure 8B), 111 items with more than five publications were screened from three clusters: 41 red, 36 green, and 34 blue. The representative journals included Prostate, the European Journal of Nuclear Medicine and Molecular Imaging, and European Urology. As shown in Table 5, a table of the top 10 main journals related to PCa bone metastasis was created. It includes journals, publications, countries, citations, and the IF/JCR partition. The most cited journal was European Urology, with 3,010 citations.

**TABLE 5:** The top 10 main journals related to PCa bone metastasis.

Rank	Journal	Number	Country	Citation	IF/JCR (2021)
1	EUR UROL	33	Netherlands	3,010	24.267/Q1
2	EUR J NUCL MED MOL I	70	Germany	2,902	10.057/Q1
3	LANCET ONCOL	12	United States	2,693	54.443/Q1
4	J NUCL MED	44	United States	2,032	11.082/Q1
5	J CLIN ONCOL	15	United States	1,637	50.717/Q1
6	PROSTATE	77	United States	1,428	4.012/Q3
7	CLIN CANCER RES	25	United States	1,419	13.801/Q1
8	ONCOGENE	33	England	1,353	8.756/Q1
9	Oncotarget	50	United States	1,337	0.000/
10	PLOS ONE	56	United States	1,195	3.752/Q2

### Bibliometric analysis of the co-occurrence

**FIGURE 9:**
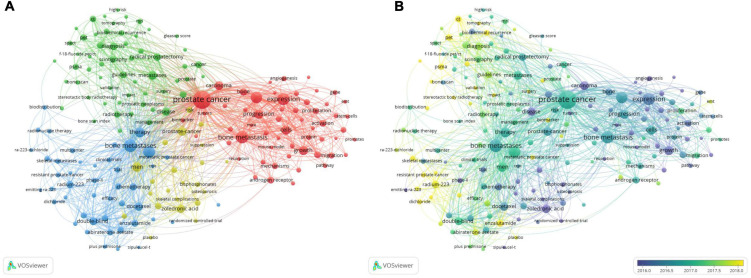
The bibliometric analysis of the co-occurrence of all keywords in the field of PCa bone metastasis. **(A)** The network visualization map of high frequency keywords in the field of PCa bone metastasis. **(B)** The overlay visualization map of high frequency keywords in the field of PCa bone metastasis.

A total of 6,753 keywords were selected that occurred more than 20 times. A network visualization map consisting of 181 high-frequency keywords was obtained (Figure 9A). Then, the keywords were divided into four categories: cluster 1: “basic research” (red), including metastasis, expression, progression, and growth; cluster 2: “assisted diagnosis and therapy” (green), including scintigraphy, prostate-specific membrane antigen (PSMA), radiotherapy, and radical prostatectomy; category 3: “clinical trials” (blue), including therapy, survival, double-blind, radium-223, chemotherapy, and phase II; cluster 4: “prognosis” (yellow), including quality of life, skeletal-related events, zoledronic acid, and risk. Among the numerous high-frequency keywords, “survival” ranked first in total link strength and second in occurrence frequency, and “expression” ranked first in occurrence frequency and second in total link strength. Therefore, these two research directions were focused on PCa bone metastasis.

The overlay visualization map (Figure 9B) shows that the focus of research has shifted from basic research to diagnosing and treating PCa bone metastasis in recent years. Regarding auxiliary examinations, precision imaging technology, such as PSMA positron emission tomography (PET)/computed tomography (CT), has become a hotspot. Radium-223 and androgen receptor (AR) antagonists are also hotspots for clinical application and research. In addition, biochemical recurrence, membrane antigen, surgery, radioligand therapy (RLT), and biomarkers of PCa bone metastasis are current research hotspots.

## Research hotspots and frontiers

Based on the above bibliometric analysis, we have summarized several research hotspots and frontiers in the study of PCa bone metastasis.

### Effects of several therapeutic methods on the survival rate and prognosis of patients with PCa bone metastasis

The treatment of PCa bone metastasis primarily includes local and systemic treatments. Local treatments include surgery and radiotherapy. The results of a study showed that the PFS was 38.6 months in the surgery group and 26.5 months in the non-surgery group under androgen deprivation treatment (ADT). In addition, the tumor-specific survival rate was significantly improved in the surgery group, whereas the OS was similar between the two groups (24).

Targeted therapy is an emerging strategy for the treatment of advanced-stage cancers (25). Radium-223 has a certain “bone targeting” feature in patients with bone metastasis. Due to its strong radioactivity, it can quickly eliminate tumor cells and causes little damage to normal bone tissue, providing it a unique advantage in the treatment of PCa bone metastasis (26). Parker et al. (20) showed that compared to placebo, radium-223 increased the median survival by 3.5 months and reduced the risk of death by 30% in metastatic CRPC (mCRPC). Sartor et al. (27) found a similar effect of radium-223 on the survival rate of patients with PCa bone metastasis. Among CRPC patients recruited with bone metastasis and no visceral metastasis, the time to first symptomatic skeletal events (SSEs) was 15.6 months in the radium-223 group compared with 9.8 months in the placebo group. In addition, patients treated with radium-233 had a reduced risk of bone pain and spinal cord compression compared to those treated with placebo. The results of these clinical trials suggest that radium-233 can be used to treat CRPC and bone metastasis.

Thus, RLT is a novel treatment for PCa bone metastasis. For example, 177Lu-PSMA-617 can accurately deliver radiation to PSMA-positive PCa cells without damaging surrounding healthy tissues. As a result, RLT effectively prolongs the OS of advanced CRPC and bone metastasis, significantly delaying the progression of disease symptoms (28–30).

ADT is the standard treatment for PCa bone metastasis (24). Enzalutamide is an AR antagonist, whereas abiraterone is an androgen synthesis inhibitor. Efstathiou et al. (31) found that the median PFS of mCRPC patients treated with enzalutamide and abiraterone was 251 days, demonstrating the safety of the combination of these two drugs. In addition, another study showed that patients with mCRPC who received abiraterone or enzalutamide as first-line treatment had a 42% reduced risk of SSEs (32). Saad et al. (33) showed that radium-233 could also be used in combination with other drugs to treat PCa bone metastasis. Patients treated with radium-233 plus abiraterone, enzalutamide, or a combination of these three drugs had a longer median OS than patients treated with radium-233 alone. In addition, patients treated with radium-233 plus denosumab had longer median OS than those treated with radium-233 monotherapy, suggesting that radium-233 can be safely used in combination with these agents. Abiraterone plus prednisone is also an effective treatment. In a phase III trial by Fizazi et al. (34), the median OS was significantly longer in the abiraterone plus prednisone group (53.3 months) than in the placebo group (36.5 months).

Regarding prognosis, zoledronic acid reduces the risk of SSEs in patients with CRPC and bone metastasis. However, early zoledronic acid use was not associated with the first occurrence of SSEs in patients with castration-sensitive PCa and bone metastasis (35). In addition, denosumab treatment significantly reduces the risk of SSEs (36). Notably, the results of a phase III clinical trial by Lipton et al. (37) showed that denosumab was superior to zoledronic acid in terms of reducing SSEs.

### Emerging diagnostic methods for biological recurrence of PCa bone metastasis

PSMA-PET is an emerging precision imaging technology. One study showed that the sensitivity and specificity of PSMA-PET in detecting bone areas were 98.8–99.0% and 98.9–100%, respectively. It also had a sensitivity of 98.7–100% and a specificity of 88.2–100% in detecting total bone accumulation. The results of these two aspects were evidently better than those of conventional diagnostic images, such as bone scans (38). In addition, the parameters of GA-68-PSMA PET, such as the PSMA reporting and data system (RADS) rating, SUVmax, and SUVmax ratio for lesions to blood pools, can improve the diagnostic accuracy of PCa bone metastasis (39). Recent studies have shown that 18F-PSMA-1007 PET/CT can be used for localizing and diagnosing biochemical recurrence of lesions after radical prostatectomy. The diagnostic rate was related to the PSA level and Gleason score (40). PSMA-PET can also be used to evaluate the grading and staging of patients with PCa (41).

In summary, PSMA-PET opens a window for PCa patients who cannot accurately identify their occurrence and metastasis. In addition, it can accurately detect, diagnose, and treat the disease, providing guidance for the further treatment of patients with recurrent PCa.

### Factors influencing the process of PCa cells metastasis to bone

miR-409-3p/409-5p is a miRNA expressed in embryonic stem cells. One study showed that it is important in promoting PCa cell growth, epithelial-mesenchymal transformation, and bone metastasis (41). These results suggest that miR-409-3p/409-5p has the potential to be a promising biomarker and target for the treatment of PCa bone metastasis. In addition, there were many types of miRNA biomarkers with good prospects, such as miR-210-3p, miR-181a-5p, miR-204-5p, and miR-133a-3p (14, 42–45).

A recent study has revealed that exosome pyruvate kinase M2 (PKM2) can promote bone metastasis by transferring PCa cells to bone marrow stromal cells. This finding suggests that PKM2 may be a biomarker and therapeutic target for PCa bone metastasis (46). Another study showed that during PCa progression, myeloid phagocytosis of apoptotic cancer cells could accelerate CXCL5-mediated bone inflammation and tumor growth (47). EGF receptors, EGFR and HER2, play important roles in the progression of many cancers. For example, EGFR has been shown to improve the survival of prostate tumor-initiating cells and circulating tumor cells to bone, while HER2 supported the growth of PCa cells at the site of metastasis (48).

Recently, it was reported that the Wnt5a/ROR2/SIAH2 signaling axis could induce and maintain the dormancy of PCa cells in the bone by inhibiting the Wnt/β-catenin signaling pathway, suggesting that the Wnt5a/ROR2/SIAH2 signaling axis may be a new therapeutic method (49). Other studies have also proposed a role for different dormancy mechanisms in PCa bone metastasis. For example, a research group found that disseminated tumor cells were induced into dormancy by TGFβ RIII-p38MAPK-pS249/pT252-RB signal in PCa bone metastasis (15). Yumoto et al. (50) revealed that a loop between the TGF-β2 signaling pathway and Gas6/Axl axis plays an important role in inducing PCa cell dormancy. Kim et al. (51) reported that TBK1 interacts with mTOR and inhibits its function in inducing PCa cell cycle arrest, which plays an important role in the dormancy and drug resistance of PCa.

## Strengths and limitations

In this study, we conducted a comprehensive and systematic bibliometric analysis of literature on PCa bone metastasis. Compared with traditional literature reviews, VOSviewer’s bibliometric analysis is more comprehensive and intuitive. However, this study has some limitations. First, the literature in our study was only extracted from the WOS core collection database, which inevitably led to the omission of other literature. Second, the document type selected was only articles published in English, which may have led to selection bias. Finally, some newly published papers of high quality may not have received much attention and are cited less frequently than classic papers.

## Conclusion

In summary, this study helps us understand the research status of PCa bone metastasis in the past decade. Bibliometric and visual analyses were used to analyze the literature worldwide. The results showed that the number of publications had an upward trend, and the United States significantly contributed to PCa bone metastasis. The journal with the highest number of publications was Prostate, and the journal with the highest number of citations was Eur Urol. Collaboration between authors, organizations, and countries/regions must be constantly strengthened. We hope that more researchers and organizations can promote academic exchanges and strengthen cooperation to continue to fill the gap in PCa bone metastasis. Countries should actively create opportunities for communication and cooperation, provide a good platform for researchers and organizations, and actively identify and solve potential problems. Factors affecting the survival rate of patients with PCa bone metastasis have been the focus of attention. Diagnosis and treatment are hot topics and may become a research trend in the future. With accurate location-based diagnosis and emerging therapies, patients with PCa bone metastasis have a better prognosis.

## Data availability statement

The original contributions presented in this study are included in the article/supplementary material, further inquiries can be directed to the corresponding author/s.

## Author contributions

KW, ZL, and XW conceived the experiments. KW, CZ, and ZL analyzed the data. KW wrote the manuscript. All authors read and approved the final manuscript.

## Abbreviations

PCa: Prostate cancer
WOS: Web of Science
IF: Impact factor
JCR: Journal citation reports
PSMA: Prostate-specific membrane antigen
PET: Positron emission tomography
CT: Computed tomography
CRPC: Castration-resistant prostate cancer
mCRPC: Metastatic Castration-resistant prostate cancer
PFS: Progression-free survival
BSI: Bone scan index
OS: Overall survival
AR: Androgen receptor
RLT: Radioligand therapy
ADT: Androgen deprivation treatment
SSEs: Symptomatic skeletal events
PKM2: Pyruvate kinase M2
RADS: Reporting and data system.
